# The Cause of Death of Col. Ingersol

**Published:** 1899-08

**Authors:** 


### THE CAUSE OF DEATH OF COL. INGERSOL.

No medical man can read the account of the death of Col. Ingersol in the daily papers without a misgiving that the large doses of a particular medicine he took, and that a most powerful one, had much to do with it.

The doctor directed him "to keep taking Nitro-glycerine tablets that he had prescribed once in fifteen minutes till the pain subsided.

"These tablets are so powerful that the instant one was swallowed it made the veins on Col. Ingersol's forehead stand out like whipcord. The medicine is given to relieve the strain on the heart by distending the blood-vessels, thus making its work lighter."

Now if the reader will turn to the pathogenesis of Nitro-glycerine or, as it is called, "Glonoine," in our materia medica, and carefully note what is there said, he will see that the drug in such powerful doses, so frequently repeated, was the cause of death, and that it was a most lamentable case of mistaken zeal to have prescribed it.

The rational explanation of the action of Nitro-glycerine given in the newspaper cutting is a most deceptive incentive to its careless use and to utter blindness to its power for evil.

This case should be a warning to all medical men who believe in giving large doses of powerful remedies and especially of Nitro-glycerine, the effects of which are little known.

